# Supplementary material for: Ferroelectric Tuning of ZnO Ultraviolet Photodetectors
Source: Nanomaterials (Basel). 2022 Sep 27;12(19):3358. doi: 10.3390/nano12193358 (PMC9565710; doi:10.3390/nano12193358)
Supplement: Supplementary file 1 [file nanomaterials-12-03358-s001.zip › nanomaterials-1918142-supplementary.pdf]

Supplementary Materials

Ferroelectric Tuning of ZnO Ultraviolet Photodetectors

Haowei Xie [†], Chenxu Kang [†], Muhammad Ahsan Iqbal, Xiaoliang Weng, Kewen Wu, Wei Tang, Lu Qi, Yu-Jia Zeng [*]

Key Laboratory of Optoelectronic Devices and Systems of Ministry of Education and Guangdong Province, College of Physics and Optoelectronic Engineering, Shenzhen University, Shenzhen 518060, China

E-mail: yjzeng@szu.edu.cn

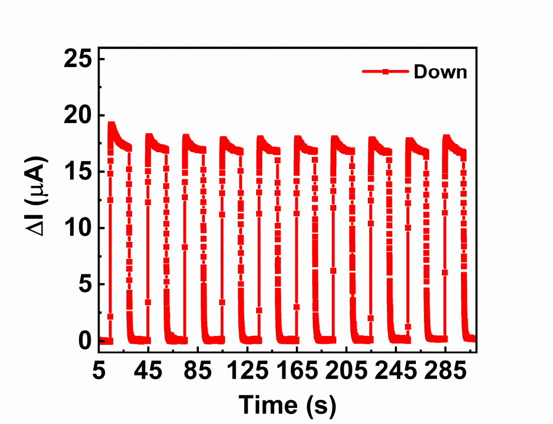

Figure S1. The ΔI-t characteristic curves of the device in the Down state when V_{DS}= 0.1 V.

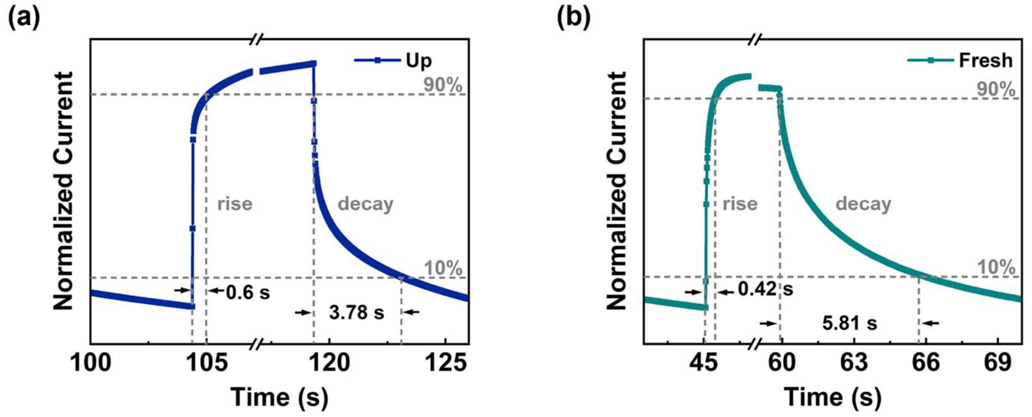

Figure S2. The response time of the ZnO NW Fe-FET device in a) the Up state and b) the Fresh state.

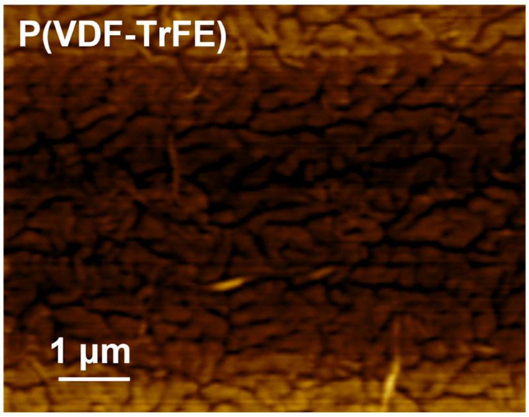

Figure S3. Atomic force microscopy (AFM) image of the P(VDF-TrFE) film.

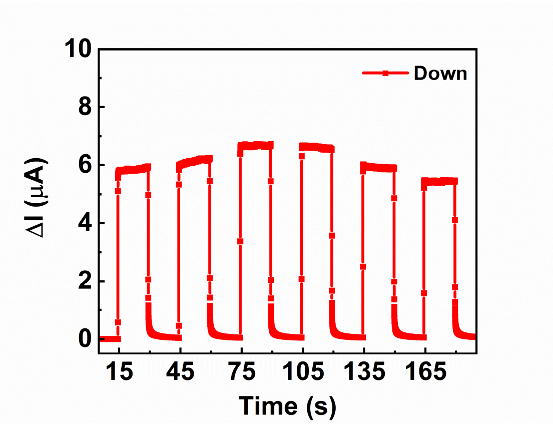

Figure S4. The ΔI-t characteristic curves of the device in the Down state when V_{DS}= 1 V.

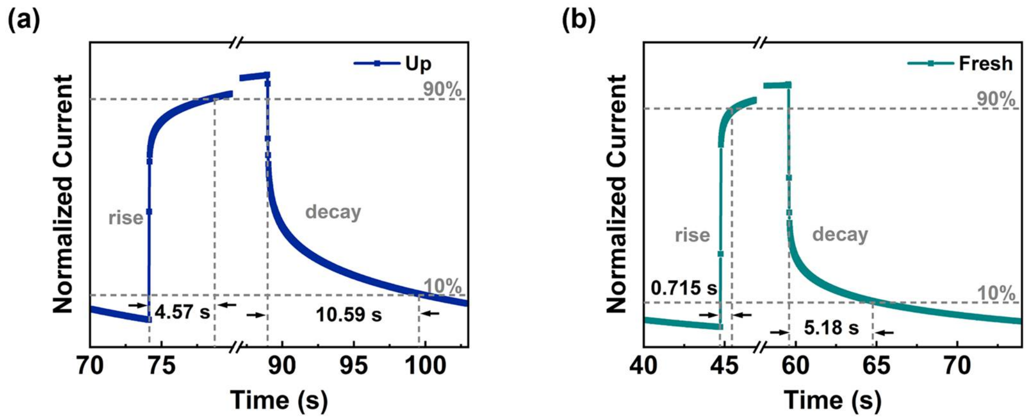

Figure S5. The response time of the ZnO film device in (a) the Up state and (b) the Fresh state.

Table S1. Comparison of the main parameters of the UV photodetectors in this work.

Materials	Rise/Fall time [ms]	Responsivity [A/W]	Bias [V]	Detectivity [Jones]	Ref.
**CuBr**	32/48	3.17	10	1.4×10^{11}	[35]
**FeS_2-PbS/graphene**	-	1.78×10^{6}	0.1	6.46×10^{11}	[36]
**$MAPbI_3/TiO_2$**	$(2/1) \times10^{3}$	1.3	1	2.5×10^{12}	[37]
**Ag-p-NiO/n-rGO**	800/840	0.072	0	3.95×10^{12}	[38]
**$CuZnS/TiO_2$**	-	640	3	-	[39]
**ZnO NWs/CIPS**	110/450	2.40×10^{4}	0.1	7.17×10^{11}	This work
